# Insights of biosurfactant producing *Serratia marcescens* strain W2.3 isolated from diseased tilapia fish: a draft genome analysis

**DOI:** 10.1186/1757-4749-5-29

**Published:** 2013-10-22

**Authors:** Xin Yue Chan, Chien Yi Chang, Kar Wai Hong, Kok Keng Tee, Wai Fong Yin, Kok Gan Chan

**Affiliations:** 1 Division of Genetics and Molecular Biology, Institute of Biological Sciences, Faculty of Science, University of Malaya, Kuala Lumpur 50603, Malaysia; 2 School of Molecular Medical Sciences, Centre for Biomolecular Sciences, University of Nottingham, Nottingham, UK; 3 Interdisciplinary Computing and Complex Systems Research Group, School of Computer Science, University of Nottingham, Jubilee Campus, Wollaton Road, Nottingham NG8 1BB, UK; 4 Centre of Excellence for Research in AIDS (CERiA), Department of Medicine, Faculty of Medicine, University of Malaya, Kuala Lumpur 50603, Malaysia

**Keywords:** *Serratia marcescens*, Biosurfactant, Serrawettin, Quorum sensing, *N*-acyl homoserine lactone, *swrA*, Next generation sequencing technology

## Abstract

**Background:**

*Serratia marcescens* is an opportunistic bacterial pathogen with broad range of host ranging from vertebrates, invertebrates and plants. *S. marcescens* strain W2.3 was isolated from a diseased tilapia fish and it was suspected to be the causal agent for the fish disease as virulence genes were found within its genome. In this study, for the first time, the genome sequences of *S. marcescens* strain W2.3 were sequenced using the Illumina MiSeq platform.

**Result:**

Several virulent factors of *S. marcescens* such as serrawettin, a biosurfactant, has been reported to be regulated by *N*-acyl homoserine lactone (AHL)-based quorum sensing (QS). In our previous studies, an unusual AHL with long acyl side chain was detected from this isolate suggesting the possibility of novel virulence factors regulation. This evokes our interest in the genome of this bacterial strain and hereby we present the draft genome of *S. marcescens* W2.3, which carries the serrawettin production gene, *swrA* and the AHL-based QS transcriptional regulator gene, *luxR* which is an orphan *luxR*.

**Conclusion:**

With the availability of the whole genome sequences of *S. marcescens* W2.3, this will pave the way for the study of the QS-mediated genes expression in this bacterium.

## Background

*Serratia marcescens* is common microorganism presence in soil and freshwater [1]. However, the emergence of multidrug resistant *Serratia* has been alarming not only in the medical field but also aquaculture and agriculture sectors [2-4]. In 2009 an endemic disease outbreak in fish farms of Malaysia had killed more than 50% of the tilapia fish. Five bacteria strains including *S. marcescens* W2.3 have been isolated from the fish samples and been suspected to be the causal agent of the outbreak.

Quorum sensing (QS) describes bacteria community gene regulation by cell-cell communication through the production of QS signalling molecule [5]. *S. marcescens* which is taxonomically classified as *Proteobacteria* produces *N*-acyl homoserine lactone (AHL) as QS signal molecules. Its AHL-based QS system plays a regulatory role in biosurfactant production, biofilm formation, motility, prodigiosin and nuclease production that contribute to the pathogenesis [1,6]. Unlike most of *S. marcescens*, *S. marcescens* W2.3 produce *N*-dodecanoyl-homoserine lactone (C12-HSL) rather than short chain AHLs. Therefore, this suggests the presence of novel AHLs responding proteins and virulence factors that may be regulated under this long chain AHL-based QS in *S. marcescens* W2.3.

The rapid maturation of next generation sequencing (NGS) technology coupling with the fast improvement of computing power enable researcher to map bacteria genome within short period of time. Annotating the draft genome with the aid of databases available allow us to look into several genes simultaneously. Here, we present insights of the *S. marcescens* W2.3 genome, describing the presence of putative QS related genes and the serrawettin coding gene, *swrA*.

## Methods

### Bacterial culture

*S. marcescens* W2.3 is routinely maintained on either LB (Luria- Bertani, BD, USA) agar plates at 37°C or culture for 20 hrs in broth at 28°C with 200 r.p.m shaking.

### Genomic DNA extraction

Genomic DNA of the bacteria was extracted with QIAamp DNA Mini kit (Qiagen, USA) and was subjected to RNase (Qiagen, USA) treatment. The DNA was eluted with elution buffer and subjected to DNA quantification with Qubit® 2.0 Fluorometer (dsDNA High Sensitivity Assay Kit) (Invitrogen, USA), and qualification with Nanodrop Spectrophotometer and agarose gel electrophoresis. The genomic DNA was stored in -20°C.

### Library preparation and sequencing

DNA sequencing template was prepared with Nextera™ DNA Sample Preparation kit (Nextera, USA). The quality of DNA library was validated by Bioanalyzer 2100 high sensitivity DNA kit prior to sequencing. Upon sequencing, DNA (6 pM) was loaded into the sequencing cartridge and the sequencing was performed on Illumina MiSeq platform.

### Read quality assessment

The quality of raw sequences as well as (G + C) content was checked with FastQC. Raw reads were trimmed at Phred 30 and were *de novo* assembled using CLC Genomic Workbench 5.1 [7]. Trimmed sequences were assembled with length fraction of 0.8 and similarity fraction of 0.8. Contigs with at least 30-fold coverage were subjected to gene prediction using Prodigal 2.6 [8].

Gene annotation was performed using RAST (Rapid Annotation using Subsystem Technology) followed by visualization of the bacterial genome using GeneWiz Browser 0.94 Server [9,10]. In addition to RAST, Serrawettin genes were annotated by BLAST against NCBInt/nr database with e-value of 0.0001 and aligned with reference genes using LAST [9,11]. Phylogenetic analysis was performed using MEGA version 5.0 [12].

## Quality assurance

The 16S rDNA gene from draft genome was used to check for contamination. RNAmmer 1.2 Server has shown that only a copy of 16S rDNA gene is presence in the draft genome. The contig that carried 16S rDNA gene was annotated by BLAST against NCBI microbial 16S database and confirmed this 16S rDNA gene belongs to *Serratia marcescens*[13,14].

## Initial findings

**Figure 1:**
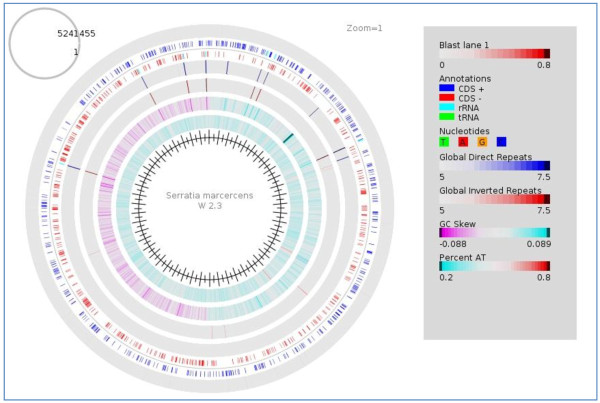
**Atlas of *Serratia marcescens *W2.3.** The atlas was constructed by GeneWiz Browser 0.94 and the GC skew was shown in the fifth lane counting from the outer most lane. The genome of *Serratia marcescens* W2.3 was 5.2 Mbps.

This sequencing generated 2,724,434 paired end reads whilst 2,509,440 quality reads were preceded to assembly after trimming. The genome size of *Serratia marcescens* W2.3 is 5.3 Mbp. The draft genome of *S. marcescens* W2.3 is made up of 72 contigs with the length of at least 500 bps with the average coverage of 60-folds (Figure 1). N50 of this assembled genome turns out to be 216,941 bp while the (G + C) content of the draft genome is 59.3%. Prodigal shows that this draft genome carried 4891 coding DNA sequence (CDS).

**Figure 2:**
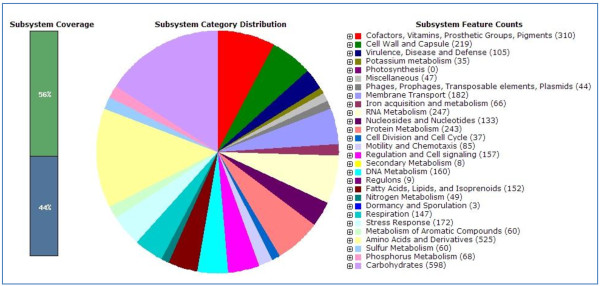
**Subsystem category distribution statistics for *Serratia marcescens *W2.3.** The pie chart present the abundance of each subsystem category and the count of each subsystem feature was listed in parentheses at the chart legend.

Draft genome was annotated using RAST and the result was shown in Figure 2. Similar with most other bacteria, most of the genes are responsible for carbohydrates and amino acid metabolism with 598 counts. This top hit follows by amino acids and derivatives, cofactors, vitamin, prosthetic groups and pigment production, RNA metabolism and cell wall and capsule synthesis. These genes are responsible for the basic needs in sustaining the life of a bacteria cell. In addition to the necessary genes, there are 105 genes responsible for virulence, disease and defense suggesting *S. marcescens* W2.3 is a pathogen.

**Figure 3:**
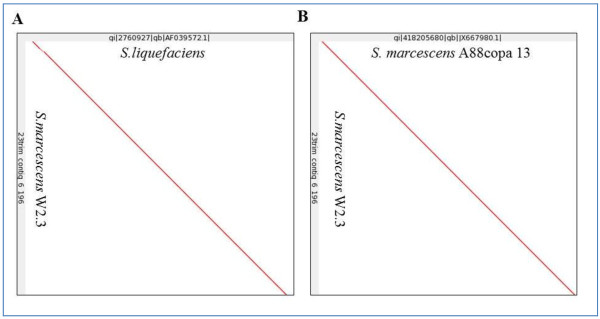
**Dotplot of *Serratia marcescens *W2.3 serrawettin, *swrA *gene DNA sequence against reference genes. (A) ***S. marcescens* W2.3 serrawettin gene is 94% similar to the *swrA* of *Serratia liquefaciens* (gb|AF039572) **(B)** and 93% similar to the *swrA* of *Serratia marcescens* A88copa 13 (gb|JX667980).

*Serratia* produces serrawettin that acts as wetting agent to reduce surface tension of the environment [15,16]. The reduction of host cell surface tension by serrawettin causes the rupture of host cell leading the success of infection by this pathogen [17]. Serrawettin gene, *swrA* was found within the genome of *S. marcescens* W2.3. This 2631 bp gene was located at contig number 6. Dotplot (Figure 3) from LAST and BLAST result show that its serrawettin gene has 94% similarity with the serrawettin gene *S. liquefaciens* (Figure 3A) and 93% similarity with the serrawettin gene from *S. marcescens* A88copa13 (Figure 3B).

In a complete AHL-based QS system, the *luxI/R* homologs interact with each other where LuxI type protein synthesis AHL and binds to the LuxR-type protein [18]. Subsequently, this AHL-protein complex regulates the expression of certain genes leading to the group behavior of the bacteria [19]. However, *luxI* and *luxR* gene do not always occurs in paired in *Proteobacteria.* For example *Pseudomonas aeruginosa* and *Sinorhizobium meliloti* have been reported to carry unpaired *luxR* gene in their genome [18,20]. These unpaired receptor protein coding genes does not responsible for any signalling molecule production but they are responsive to the cognate signalling molecules produced by both its existing AHL synthase and the signalling molecules from the environment [21].

**Figure 4:**
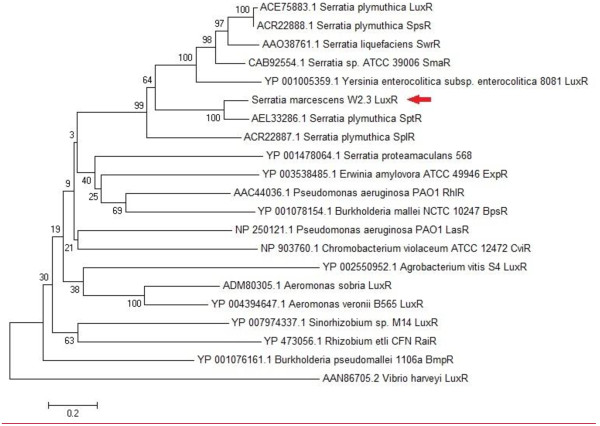
**Phylogenetic analyses of *S. marcescens luxR *gene.** The tree was constructed based on the *luxR* protein sequences by Neighbor-Joining with bootstraps value of 1000 replicates.

A putative *luxR* gene with the size of 759 bps was identified within contig 7 of *S*. *marcescens* W2.3 draft genome. Phylogenetics analysis of the LuxR protein sequence of *S. marcescens* strain W2.3 and its closely related species shows that it is highly similar to the LuxR protein sequence of *S. plymuthica* (SptR) (Figure 4). However, no *luxI* gene was identified within the upstream and downstream of this *luxR*, thus we hypothesised that this is an orphan *luxR.* In 1998, Cox *et al* reported a solo *luxR* of *S. marcescens*, *carR* that regulates its carbapenem production [22]. However, the unpaired *luxR* gene of *S. marcescens* W2.3 is not group into the same transcriptional protein family as *carR* gene; therefore more studies need be conducted in the future for further understanding of the interaction of unusual long AHL with *luxR* coding in the genome of *S. marcescens* W2.3 isolated from the diseased tilapia fish and its rule in the pathogenesis*.*

## Future directions

The whole genome of *S. marcescens* W2.3 has shown the presence of virulence factor coding genes and we have found the complete sequence of the serrawettin synthase gene. Since serrawettin production of *S. marcescens* is reported to be coordinated by QS, our future work will be focusing on the study of the AHL­based QS gene regulation of *S. marcescens* W2.3.

## Availability of supporting data

This whole genome shotgun project has been deposited at DDBJ/EMBL/GenBank under the accession ALOV00000000. The version described in this paper is the first version ALOV01000000.

## Abbreviations

AHL: *N*-acyl homoserine lactone; QS: Quorum sensing; PBS: Phosphate buffer saline; LB: Luria bertani; CDS: Coding DNA sequence; NGS: Next generation sequencing; RAST: Rapid annotation using subsystem technology; C12HSL: *N-*dodecanoyl homoserine lactone.

## Competing interests

The authors declare that they have no competing interests.

## Authors’ contributions

XYC and KWH performed the DNA sequencing assay. XYC, CYC, KWH, WFY and KGC analyzed the sequencing data. XYC, CYC, KKT and KGC contributed to the writing of the manuscript. All authors read and approved the final manuscript.
